# Protein misfolding in neurodegenerative diseases: implications and strategies

**DOI:** 10.1186/s40035-017-0077-5

**Published:** 2017-03-13

**Authors:** Patrick Sweeney, Hyunsun Park, Marc Baumann, John Dunlop, Judith Frydman, Ron Kopito, Alexander McCampbell, Gabrielle Leblanc, Anjli Venkateswaran, Antti Nurmi, Robert Hodgson

**Affiliations:** 1 Discovery Services, Charles Rivers Laboratories, Wilmington, MA USA; 2 Royal Veterinary College, University of London, London, UK; 3 Health & Life Science Consulting, Los Angeles, CA USA; 4 Biochemistry and Developmental Biology, University of Helsinki, Helsinki, Finland; 5 Neuroscience Innovation Medicines, Astra Zeneca, Cambridge, MA USA; 6 Stanford University, Stanford, CA USA; 7 Neurology, Biogen Idec, Cambridge, MA USA; 8 Leblanc Bioscience Consulting, Berkeley, CA USA

**Keywords:** Neurodegeneration, Proteostasis, Mouse models, Biomarkers, Chaperones, Drug discovery

## Abstract

A hallmark of neurodegenerative proteinopathies is the formation of misfolded protein aggregates that cause cellular toxicity and contribute to cellular proteostatic collapse. Therapeutic options are currently being explored that target different steps in the production and processing of proteins implicated in neurodegenerative disease, including synthesis, chaperone-assisted folding and trafficking, and degradation via the proteasome and autophagy pathways. Other therapies, like mTOR inhibitors and activators of the heat shock response, can rebalance the entire proteostatic network. However, there are major challenges that impact the development of novel therapies, including incomplete knowledge of druggable disease targets and their mechanism of action as well as a lack of biomarkers to monitor disease progression and therapeutic response. A notable development is the creation of collaborative ecosystems that include patients, clinicians, basic and translational researchers, foundations and regulatory agencies to promote scientific rigor and clinical data to accelerate the development of therapies that prevent, reverse or delay the progression of neurodegenerative proteinopathies.

## Background

Many neurodegenerative diseases involve the misfolding and aggregation of specific proteins into abnormal, toxic species. Therapeutic targeting of protein misfolding has generated unique challenges for drug discovery and development for several reasons, including 1) the dynamic nature of the protein species involved, 2) uncertainty about which forms of a given disease protein (monomers, oligomers, or insoluble aggregates) are primarily responsible for cellular toxicity, 3) our still limited understanding about which components of the cellular proteostatic machinery these disease proteins interact with and 4) lack of well-validated biomarkers for clinical trials. However, as we continue to gain knowledge of disease mechanisms, improve our abilities to model disease states in vitro and in vivo, and identify new biomarkers, there is increasing optimism that we will discover novel therapeutics that prevent, reverse, or delay the progression of neurodegenerative diseases. In concert with the scientific advances in the past several decades, the field of neurodegenerative disease research is undergoing significant change with respect to how various stakeholders engage each other and share information with the entire community. Increasing collaboration between scientists from the pharmaceutical industry disease foundations, academic researchers, contract research organizations, and patient advocacy group, and increasing communication between groups studying different diseases, has spurred promising initiatives in basic, translational, and clinical research in neurodegenerative disease.

**Fig. 1:**
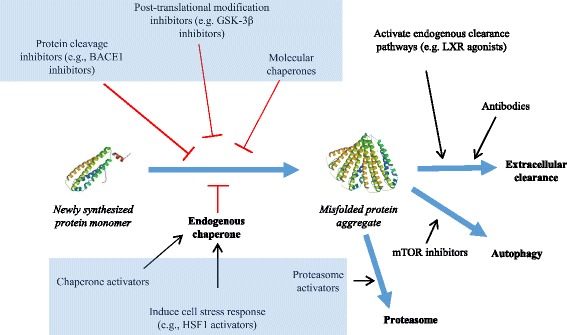
Mechanisms involved in protein misfolding & therapeutic targets. A newly synthesized protein is stabilized by endogenous chaperone proteins. Under normal conditions abnormal protein aggregates (misfolded proteins) are degraded and/or cleared extracellularly, undergo autophagy or are degraded with the aid of the cellular proteasome. In cases of abnormality and misfolding of proteins (such as those present in many neurological diseases) post translational modification inhibitors, protein cleavage inhibitors and extrinsic molecular chaperones have been used in attempts to curtail or correct protein misfolding. In addition, post translational approaches to address and combat the presence of misfolded proteins include agonists that attempt to activate endogenous clearance pathways as well as the introduction of recombinant antibodies to work against the rogue protein

There are multiple steps in the production and processing of disease proteins that could be targeted therapeutically, from initial synthesis to degradation and extracellular clearance (Fig. 1). This review discusses the advantages and potential problems associated with targeting different pathways involved in the production and processing of misfolded proteins, and highlights new candidate therapeutics that have been developed by targeting specific steps in the life cycles of disease proteins. It also discusses some key issues involved in translating preclinical findings to successful clinical trials^1^.

## Roles of misfolded proteins and aggregates in proteinopathies

Misfolded proteins exist in cells together with unfolded, intermediately folded, and correctly folded species [1]. In healthy cells, misfolded proteins are either degraded or refolded correctly by chaperone proteins that are involved in protein folding and trafficking as well as intermediate stabilization [1]. Indeed, it is now believed that many, if not all, proteins can form amyloid fibrils under appropriate biochemical conditions [2, 3]. However, many disease-associated amyloidogenic proteins have extensive regions of intrinsic disorder in their free soluble forms [4] and have specific, often short, internal amino acid sequences that are necessary and sufficient to support aggregation [3, 5]. These same motifs can be found in other non-disease proteins, and when liberated from rest of protein these fragments will aggregate into cytotoxic amyloid fibrils [2, 5].

Once formed, higher order amyloid aggregates are highly resistant to degradation. Proteasomes can degrade only single chain polypeptides, and also require the proteins to be partially or fully (in the case of proteasomes) unfolded [6]. In addition, the amyloid state is extremely stable thermodynamically, because of the extensive contacts made between the protein chains of the polymer. The thermodynamic stability of amyloid aggregates also contributes to their ability to convert native proteins into amyloid forms (i.e., to seed prion-like propagation) [7].

Under conditions of proteotoxic stress, cellular aging, or the presence of disease mutations, proteins can escape a cell’s quality control system and begin to aggregate into non-native structures, which range from oligomers and amorphous assemblies to highly ordered amyloid fibrils and plaques.

Cells are normally faced with a continuous stream of misfolded proteins arising from mistakes in biogenesis, disease-causing mutations, and physiological stressors (Table 1 lists misfolded proteins associated with neurodegenerative disease). They deal with misfolded proteins by refolding, degrading, or sequestering them in specific intracellular compartments, such as aggresomes or other types of inclusion bodies. Chaperone proteins bind to nascent polypeptides as they emerge from ribosomes and assist in their folding, and oversee and participate in every step in the handling of misfolded proteins. Chaperones also monitor the quality of the folded chains and can in some cases unfold and refold misfolded proteins. Alternatively, chaperones target the misfolded proteins for degradation via the ubiquitin proteasome system or autophagy pathway, or for sequestration in various cellular compartments [8, 9].

**Table 1:** Misfolded proteins associated with neurodegenerative diseases

Proteinopathy	Aggregating protein(s)
Alzheimer’s disease	Amyloid beta (Ab) peptide; Tau
Parkinson’s disease	α-synuclein
Multiple tauopathies	Tau protein (microtubule associated)
Huntington’s disease	Huntingtin with tandem glutamine repeats
Amyotrophic lateral sclerosis	Superoxide dismutase 1
Spongiform encephalopathies	Prion proteins
Familial amyloidotic polyneuropathy	Transthyretin (mutant forms)

Studies in yeast have revealed two overlapping but functionally distinct networks of chaperones [10, 11]. CLIPs (chaperones linked to protein synthesis) are physically associated with the translational machinery and oversee quality control of newly translated proteins. CLIPs comprise a large family of proteins, and evidence suggests that different CLIPs associate with different classes of proteins [12]. The second set of chaperones, heat shock proteins (HSPs), protects the proteome from denaturing environmental stressors, including thermal, oxidative, and hypoxic stresses. CLIPs and HSPs have different modes of transcriptional regulation in yeast: CLIPs are generally down-regulated under conditions of proteotoxic stress, whereas HSPs are up-regulated [9, 10]. Subnetworks of differentially regulated chaperones and co-chaperones have also been identified in *C. elegans* and in the human brain [13]. In addition, it has been found that as the human brain ages, a subset of chaperones consisting primarily of CLIPs are repressed, and chaperones that help protect the proteome against misfolded protein toxicity are induced mimicking proteotoxic stress; these differences are even more pronounced in the brains of people with Alzheimer’s, Huntington’s, or Parkinson’s disease [13]. Misfolded proteins that are not immediately refolded are actively sequestered in spatially and functionally segregated quality control compartments [8, 14]. In yeast, the juxtanuclear quality control (JUNQ) compartment concentrates soluble misfolded proteins that are either later refolded by chaperones or degraded by the ubiquitin proteasome system (UPS). The insoluble protein deposit (IPOD) compartment, which may be equivalent to the aggresomes found in mammalian cells, sequesters insoluble aggregates. The sequestration of aggregated misfolded proteins may in many cases serve a beneficial role – by preventing misfolded proteins from saturating chaperones and proteasomes, facilitating their clearance via the UPS or through autophagy, or by preserving them for subsequent refolding and return to use in the cell [15, 16].

### Proteostasis

The term “proteostasis” refers to the integrated activity of cellular mechanisms that regulate protein production, folding, trafficking, degradation, and clearance. Cellular responses to proteotoxic stress, like the heat shock response and the unfolded protein response (UPR) involve large-scale rebalancing of the proteostatic network via transcriptional regulation of both chaperones (e.g., Hsp70, Hsp90) and non-chaperone proteins (including transcription factors, signaling proteins and receptors, and cell cycle regulators [17]. Post-translational modifications can also radically change the activity of some chaperones [18], and likely also play a key role in proteostasis, although this area remains largely unexplored. During the ageing process, or in diseases associated with misfolded proteins, cells may experience “proteostatic collapse.” Proteostatic collapse is associated with the accumulation of ubiquitinated inclusion bodies (IBs), which are seen in many neurodegenerative diseases [9]. It has been suggested that ubiquitinated aggregates can directly inhibit or clog proteasomes [19, 20]. However, in the case of ubiquitinated Huntingtin (Htt), this does not appear to be the case, nor is ubiquitination required for Htt to accumulate in IBs [21]. Rather, the accumulation of ubiquitinated species in misfolded protein diseases may reflect a global perturbation of proteostasis, in which chaperones and proteasomes are simply overwhelmed with client proteins.

### Propagation

A key feature of misfolded protein diseases is the ability of the pathogenic protein species to propagate in a prion-like manner by recruiting normally folded counterparts to adopt pathogenic conformations. Pathogenic amyloids can also spread from neurons to other neurons and neighboring glia to initiate new pathology after injection into the brains of normal animals [22, 23]. Both in vivo and in vitro studies have shown that misfolding of one disease causing protein can induce misfolding of other aggregation-prone proteins [23], and aggregates of different disease proteins may be found in the same patient [24]. Moreover, the accumulation of one species of misfolded proteins can impair the entire proteostatic network, thereby triggering the misfolding of unrelated proteins that would otherwise fold normally [25, 26]. The mechanisms by which misfolded proteins spread from one neuron to another are currently an area of active investigation. New evidence suggests that inter-neuronal spread of misfolded proteins involves 1) activity-dependent secretion by exosomes ([27] and/or 2) chaperone-mediated pathways [28, 29].

### Mechanisms of misfolded protein toxicity

In the long term, all neurodegenerative disease proteins produce synaptic dysfunction and loss and, ultimately, neuronal cell death. The precise upstream mechanisms by which different misfolded disease proteins cause neurotoxicity are still unclear, and appear to differ depending on the protein species involved. Misfolded disease proteins appear to act primarily by toxic gain-of-function and/or dominant-negative effects, although loss-of-function effects have also been observed. Direct, acute effects of misfolded proteins on neuronal function have been observed after treating neurons with purified oligomers or transfecting them with expression vectors. To give just a few examples, amyloid-beta, tau, and alpha-synuclein all interfere with synaptic signaling [30–32]; mutant tau disrupts microtubule function and neuronal transport mechanisms [32, 33]; and alpha-synuclein disrupts mitochondrial protein import [32, 34]. In addition, larger aggregates of misfolded proteins may exert toxic effects by binding to and sequestering other cytosolic proteins. For example, proteomic studies of artificial proteins designed to form amyloid-like fibrils showed that the toxicity of these proteins correlated with the ability of their aggregates to engage in aberrant protein interactions and disrupt the cytosolic stress response [35]. Notably, the endogenous cellular proteins sequestered by the amyloid aggregates tended to be relatively large in size and enriched in intrinsically unstructured regions, and many play key roles in essential cellular activities such as transcription, translation and protein quality control. Indeed, another emerging common feature among misfolded disease proteins is their ability to disrupt proteostasis (see more below). More recently, cytosolic aggregates of several different proteins, including artificial β-sheets, fragments of mutant huntingtin, and TAR DNA binding protein-43 (TDP-43) have also been shown to disrupt nucleocytoplasmic transport of both proteins and RNA [36].

In addition to synaptic dysfunction, other cellular changes common to the major neurodegenerative diseases include calcium signaling abnormalities, mitochondrial dysfunction, oxidative stress, and neuroinflammation. These symptoms of cellular distress often occur early in the disease process, and are believed to be a cause as well as a consequence of neurodegeneration. That is, the relationship between the accumulation of misfolded disease proteins and other signs of cellular distress is bidirectional, and in many cases mutually exacerbating. For example, amyloid-β, a-synuclein, and mHtt all cause acute oxidative stress in neurons and/or astrocytes, and impair astroglial anti-oxidant responses [37–40]. Conversely, oxidative stress promotes the aggregation of disease proteins, and contributes to age- and disease-related proteostatic collapse [41, 42]. Similarly, there appearaas a downward spiralling cycle of interactions between protein misfolding and neuroinflammation, which has been most extensively studied for AD. Soluble Aβ oligomers and insoluble Aβ aggregates have been shown to bind to and activate and microglia and astrocytes, stimulating a chronic low level state of neuroinflammation [43]. Several lines of evidence suggest that the pro-inflammatory effects of Aβ, while perhaps helpful in the short-term, ultimately impair the microglial and astroglial function, including their ability to dispose of Aβ and other misfolded proteins [38, 43–45]. The destructive consequences of the neuroinflammation provoked by misfolded disease proteins are likely exacerbated by ongoing, age-related senescence of the immune system senescence [46, 47].

## Therapeutic targets

### Targeting production, misfolding and aggregation

The development of drugs targeting protein misfolding or aggregation has been challenging due to the lack of certainty about which form/s of a given disease protein is primarily responsible for the disease. In the case of amyloid-β (Aβ), it was originally thought that fibrils and plaques were the pathogenic species in Alzheimer’s disease, but more recent studies point to aggregation intermediates (oligomers and soluble protofibrils) as the primary culprits; similar findings have emerged with respect to different species of α-synuclein in Parkinson’s disease [4, 48]. The situation is further complicated by the existence of the variety of intermediate species that exist during the folding and oligomerization processes. Recent studies have demonstrated that aggregated fibrils of tau, α-synuclein, and Aβ exist in different conformational variants, or ‘strains’, that have different propagation properties and different levels of neurotoxicity [49–51].

Identifying key toxic species of misfolded proteins has been challenged the inability of conventional biochemical analytic methods to detect and characterize intermediate species. For example, denaturing gel electrophoresis (SDS-PAGE) has been shown to alter the oligomerization state of Aβ42 oligomers [52]. Recently, ion mobility spectrometry-mass spectrometry (IMS-MS) and nuclear magnetic resonance (NMR) spectroscopy have been used to analyze the folding and aggregation of amyloid proteins in solution and to identify inhibitors of these processes [53–55]. As an alternative to preventing the initial misfolding and aggregation of amyloids, another approach now being explored is to stabilize mature fibrils to prevent their prion-like propagation [48, 56].

In cases where the relative pathogenicity of various misfolded protein species is unknown, one strategy would be to intervene therapeutically as far upstream as possible in the protein synthesis pathway: i.e., at the level of protein translation, cleavage, or post-translational modification. In theory, targeting early steps in the processing pathway would provide the highest degree of therapeutic specificity and eliminate toxic gains or losses of function caused by misfolded or aggregated forms of the protein, while preventing the propagation of abnormal folding and aggregation. Protein cleavage and post-translational modification targets are being actively explored for Aβ using Beta Amyloid Cleaving Enzyme (BACE) inhibitors (some of which are now in clinical trials) and for tau using inhibitors of tau phosphorylation (e.g., glycogen synthase 3β inhibitors) and acetylation [57–60].

### Targeting chaperones

Chaperones are another possible target class for therapeutic intervention in protein misfolding disease states. As chaperones are involved in all aspects of proteostasis, they offer potential therapeutic entry points to each step in the processing of a pathogenic protein. There are over 200 different chaperone proteins expressed in the mammalian brain [61], and different cell types express different sets of chaperones and co-chaperones [62, 63]. Cell type-specific expression of chaperone subsets may help to explain why some misfolded proteins are toxic in one cell type and not in others, and also presents opportunities to develop drugs targeting neuron- or glial cell-specific chaperones. However, the sheer number of chaperones and the diversity in their mechanisms of action also presents challenge to therapeutics development; we still have limited knowledge of which chaperones interact with which disease proteins and how. Some clues have been offered by links between mutations in specific chaperones and hereditary forms of certain neurodegenerative diseases. For example, mutations in Hsp70 and Hsp40 have been linked to Parkinson’s disease [64, 65], and mutations in the co-chaperone valosin-containing protein (VCP) have been found in ALS [66]. In addition, to date over 20 different chaperones have been found to confer neuroprotection when over-expressed in cell or animal models of various neurodegenerative diseases, and in many cases individual chaperones appear to protect against several different disease proteins [67].

One approach to therapeutically targeting the chaperone system has been to develop small molecule inhibitors or activators of specific chaperones. Among chaperones relevant to neurodegeneration, Hsp70 and Hsp90 have been the most intensely studied. Hsp70 and Hsp90 have opposing effects on client protein stability: Hsp70 promotes their degradation via the UPS system, whereas Hsp90 stabilizes client proteins and inhibits their ubiquitination. The activities of Hsp90 and Hsp70 are tightly linked via HSF1. Hsp90 inhibitors typically activate HSF1, which in turn induces Hsp70 [68]. A variety of small molecule drugs have been developed that inhibit Hsp90, activate Hsp70, or both, and have been shown to reduce the formation of disease protein aggregates, reduce cellular toxicity, and improve neurological phenotypes in cellular and animal models of SBMA, HD, PD, and AD [62, 67, 69–71]. None of these drugs have yet entered clinical trials for use in neurodegenerative diseases, due to issues of low brain penetration and/or peripheral cytotoxicity, but active effort in this area is continuing [70, 72–74].

A second approach to developing chaperone-based therapeutics has been through protein engineering. For example, it was discovered that the yeast disaggregate Hsp104 has the ability to dissolve in vitro fibrils formed from a variety of neurodegenerative disease proteins, including tau, polyglutamine, Aβ42, α-synuclein and prion protein [75, 76]. However, relatively high concentrations of Hsp104 are needed to dissolve these proteins. Making small changes in Hsp104′s sequence yielded proteins with much higher dissaggregase activity and lower toxicity, including variants that reduced neurodegeneration in a *C. elegans* model of PD [77]. Other chaperones to which this approach might be applied include the yeast chaperonin Tric, which has the unusual ability to cross cell membranes and has been shown to protect against Htt toxicity [16], and metazoan chaperones known to have disaggregase activity (e.g., Hsp110, Hsp70, and Hsp40) [78].

### Targeting degradation

Defects in both the UPS and autophagy pathways of protein degradation are often seen in neurodegenerative diseases [79, 80]. For example, many of the gene mutations that cause familial PD encode proteins involved in the UPS and/or autophagy, including PINK-1, Parkin (a ubiquitin ligase), UCH-L1 (Ub carboxy terminal hydrolase L1), DJ-1 (PARK7), and LRRK2/PRAK8 [79, 81, 82]. As with the chaperone system, choosing promising drug targets from the UPS or autophagy pathways is challenging because of the number of proteins involved (there are ~ 500 to 1000 associated just with the UPS system). In addition, for most diseases it isn’t known which form/s of the disease protein are primarily responsible for cellular toxicity. This issue is critical when targeting protein degradation, because the pathway by which a given protein is degraded (e.g., UPS versus autophagy) can vary depending on whether the protein is in the soluble or fibrillar state, and on what specific post-translational modifications it bears [83, 84].

A number of small molecules have been identified that upregulate components of the UPS, promote the degradation of disease proteins and in vitro, and (in some cases) have neuroprotective effects on cultured cells [84–86], but few have yet been shown to be effective in vivo. One interesting exception is rolipram, an agent that stimulates the phosphorylation and activity of the 26S proteasome. Myeku and colleagues [21] showed that 26S proteasome function is impaired in a mouse tauopathy model, and that treating the mice with rolipram treatment improved 26S proteasome function, decreased tau aggregation, and improved cognition.

Inhibition of the mammalian target of rapamycin (mTOR) pathway has proven to be an exceptionally effective approach for stimulating the degradation of neurodegenerative disease proteins. mTOR, a serine/threonine kinase, is a signaling nexus that collects information about ambient levels of resources necessary for cell growth (e.g., nutrients, ATP, growth factors, and oxygen) and up- or downregulates protein synthesis and degradation accordingly. When growth conditions are favorable, mTOR inhibits autophagy by inhibiting the ULK1 complex, which is required for the biogenesis of autophagosomes. Thus, mTOR inhibitors typically have the net effect of stimulating autophagy [87, 88].

Rapamycin and other mTOR inhibitors increase the clearance of abnormal protein aggregates and slow neurodegeneration in both cell and animal models of a variety of neurodegenerative diseases, including AD, PD, spinocerebellar ataxia type 3, and frontotemporal dementia [87, 88]. In most of these cases, mTOR inhibitors have been shown to act at least in part via stimulation of autophagy. A recent study showed that in yeast, inhibiting mTOR also produces a rapid, coordinated upregulation of proteasomes and their 19S regulatory chaperones [89]. Thus, the efficacy of mTOR inhibitors in clearing a variety of neurodegenerative disease proteins may be due to the ability of these drugs to upregulate both the proteasomal and autophagic routes of protein degradation.

Several mTOR-dependent activators of autophagy, including the natural compounds curcurmin and resveratrol, are currently in clinical trials for treating neurodegenerative diseases. However, mTOR is a multifarious protein that regulates many cellular processes in addition to protein degradation, and in clinical trials to date wmTOR inhibitors have caused unpredictable and undesirable side effects. Autophagy can be stimulated mTOR-independent mechanisms [79, 87, 88], and a number of compounds, including FDA-approved drugs, have now been shown to stimulate clearance of abnormal proteins and confer protective effects in cell or animal models of neurodegenerative disease [87, 88, 90, 91]. It has been suggested that these compounds might be used in conjuction with mTOR inhibitors to maximize therapeutic benefit and minimize side effects [87, 90].

### Targeting extracellular clearance

One of the best-explored examples of targeting clearance of misfolded proteins has been the use of antibodies to promote clearance of Aβ. These antibodies are thought to operate by either or both of two mechanisms: 1) by penetrating the blood brain barrier (BBB) to bind Aβ in the extracellular space, and 2) through a”peripheral sink” effect [92]. Almost all misfolded proteins show some extracellular leakage [93], and an advantage of targeting extracellular misfolded proteins is that it can theoretically be accomplished by highly selective antibodies. For example, monoclonal antibodies have been developed to Aβ and α-synuclein that show >1000-fold higher affinities for the oligomeric versus monomeric forms of the proteins [94–96].

Current challenges in the use of Aβ antibodies include low rates of BBB penetration [97], nonspecific engagement of Aβ and uncertainty as to which antibodies engage clinically relevant forms of Aβ [98–100]. Detailed structural studies of how different antibodies interact with specific epitopes in the Aβ molecule are now underway [101] and help inform antibody design and epitope-targeting in the future. An alternative method of improving the clearance of toxic misfolded species is to harness endogenous mechanisms of protein clearance into the extracellular space. For example, LXR beta receptor agonists, which promote clearance of Aβ into the extracellular space by the ABCA1 transporter, have shown therapeutic effects in AD mouse models [102, 103]. Yet another potential avenue for new therapies targeting extracellular clearance of disease proteins is the use of non-antibody scaffold drugs [104].

### Rebalancing the proteostatic network

The ability of the cell’s proteostatic machinery to counter proteotoxic stressors deteriorates with age, and is further compromised by mutations and other disease conditions that lead to the accumulation of misfolded proteins [16]. Thus, another potential approach to the development of therapeutics would involve large-scale rebalancing of the proteostatic network. Indeed, the efficacy of mTOR inhibitors may reflect their ability to provoke large-scale rebalancing of protein synthesis and degradation pathways. Another attractive target in this regard is heat shock factor 1 (HSF1), a transcriptional activator that helps coordinate the heat shock response. The heat shock response (and other proteotoxic stress responses) diminish with age and in neurodegenerative disease [105]. In addition, it was recently shown that HSF1 degradation is abnormally elevated in mouse and human α-synucleinopathy [106]. Over-expression of human HSF1 has been shown to be neuroprotective in cell models of neurodegenerative diseases [107, 108], to reduce polyglutamine aggregate formation and prolong lifespan in a mouse model of HD [17] and to reduce pathogenic androgen receptor accumulation and neurotoxicity in a mouse model of spinobulbar muscular atrophy [109]. Small molecule activators of HSF1 have now been identified and shown to have neuroprotective effects in cell or animal models of neurodegenerative diseases [107, 108].

Agents targeting HSF1 or other master regulators of the proteostatic network have the advantages of being fast-acting and relatively agnostic to the identities of the misfolded proteins involved in a given neurodegenerative disease and to their mechanisms of aggregation and toxicity. The effects of such drugs are hard to predict, however. For example, the induction of the heat shock response actually exacerbates Htt IB formation in a cellular model of HD [110]. In addition, it has been pointed out that, under normal physiological conditions, the heat shock response and other proteotoxic stress response pathways are activated only transiently, and that multiple cellular mechanisms are in place to limit and down-regulate these responses [111]. Consistent with those facts, an Hsp90 inhibitor that induces the heat shock response in HD model mice was found to provide short-term beneficial effects, but those benefits proved transient [112]. Yet another potential issue with HSF1 activators is that HSF1 promotes tumorigenesis and is activated in a broad range of highly malignant human cancers [113, 114]. This issue is not necessarily unsurmountable, however, as it has also been shown that the HSF1 drives a different transcriptional program and stimulates different sets of cellular processes in cancer cells (including proliferation, invasion, and metastasis) than it does in normal cells [113]. The ability of HSF1 to activate distinct transcriptional programs in cancer cells versus normal cells is thought to result in part from differences in post-transcriptional modifications to HSF1 in the different cell types [113, 114], which in turn raises the possibility that the neuroprotective effects of HSF1 could be harnessed separately from its tumorigenic ones.

## Challenges in translating preclinical findings to clinical trials for diseases associated with misfolded proteins

The misfolded protein neuropathies have proven an exceptionally challenging arena for therapeutics development. Promising candidates for Alzheimer’s disease, Parkinson’s disease, ALS, and Huntington’s disease have been identified in preclinical studies, but very few have shown significant benefits in clinical trials (Table 2 lists known drug-target interactions for neurodegenerative diseases associated with misfolded proteins). This “failure to translate” has plagued the development of therapeutics for neurological diseases in general, and likely reasons for it have been discussed in detail elsewhere [115–117]. A leading cause is the lack of robust targets whose modulation results in a therapeutic benefit. The uncertainties about which process or protein to target, and resulting failures to demonstrate target engagement, result in preclinical studies in animal models that do not have predictive validity. Additionally, there is a strong need to identify translatable biomarkers in animal models for clinical studies. Finally, pharmacokinetics and drug safety pose significant challenges to successful drug development for misfolded protein diseases. Recently, however, strides have been made in the area of Alzheimer’s disease with structural studies of how antibodies interact with specific epitopes on the amyloid-β molecule, and how these interactions correlate with clinical outcomes [101].

**Table 2:** Drug-target pairs for neurodegenerative diseases associated with misfolded proteins

Compound name	Company	Disease indication	Mechanism of action	Status
TRx0237	TauRx Therapeutics	Alzheimer’s disease	Tau aggregation inhibitor	Phase II clinical trials completed
AADvac1	Axon Neuroscience SE	Alzheimer’s disease	Active tau based immunotherapy	Phase I clinical trials completed
ACI-35	AC Immune AG	Alzheimer’s disease	Phospho-tau vaccine	Phase I trial active
Arimoclomol	Orphazyme ApS	Amyotrophic Lateral Sclerosis	HSP activation	Phase II/III active
Nuedexta	Avanir Pharmaceuticals	Amyotrophic Lateral Sclerosis – PBA symptom treatment	Unknown for PBA treatment; NMDA receptor antagonist	FDA approved
Deferiprone	Generic	Parkinson’s disease	Iron chelator	Phase II recruiting
Istradefylline	Kyowa Hakko Kirin	Parkinson’s disease	Adenosine A2A receptor antagonist	Approved in Japan; no FDA approval

Another area of opportunity is the identification of novel targets. For example, the area of cell type-specific targets has been relatively unexplored. A key feature of the proteinopathies is that the proteins involved are typically expressed in many or all cell types, but cause pathological phenotypes only in specific sets of neurons. Thus, informed development of therapeutics should include understanding not only of the species of misfolded proteins involved, but also of how they affect different populations of neurons. A recent genomic/proteomic study in HD model mice identified striatum- and cortex-specific transcription modules whose expression correlated strongly with both CAG repeat length and age [118]. Interestingly, striatal modules included genes involved in establishing and maintaining medium spiny neuron identity. Another study showed that the degeneration of different subtypes of neurons (e.g. striatal versus motor neurons) is mediated by down-regulation of different sets of ER chaperones [119].

The use of transgenic rodent models to study pathogenic mechanisms presents more challenges in the case of the proteinopathies than in other diseases. The selection of the correct transgenic protein target is essential to develop a useful rodent model of overexpression. Transgenic models of disease are developed on the assumption that increased production of a particular protein drives disease development. When the correct protein target is not selected, the model does not accurately represent the pathogenic mechanism. Rodents differ from humans with respect to basic biology (i.e., glia to neuron ratio, anatomy of the brain vasculature) and the biochemical properties of misfolded disease protein aggregates [120]. A potential solution to the latter issue would be the use of human/mouse chimeras. For example, it has been shown that mutant huntingtin-expressing human glial precursor cells can impart the HD disease phenotype when grafted into the striata of normal mice, and that normal glial precursors can rescue certain phenotypes and slow disease progression when grafted into R6/2 HD mice [121].

Finally, there is a need for translatable biomarkers that robustly track the progression and severity of the disease for successful clinical trial. One approach has been to measure amounts of soluble disease protein in the cerebrospinal fluid (CSF). When analyzing soluble proteins, the stability and kinetics of protein turnover in the CSF must be established, along with inter- and intra-subject variability. A challenge with the use of soluble proteins as CSF biomarkers is their low concentrations in the CSF, which in turn produces a low signal-to-noise ratio. A new approach has been to study other components of the CSF, such as exosomes. Exosomes are released by most cell types, and carry cargoes of proteins that include misfolded disease proteins [122]. For chaperone or proteasome targets, which are intracellular, it may be necessary to develop surrogate markers to assess target engagement.

Molecular imaging approaches (e.g., positron emission tomography, or PET) are an alternate approach to assessing target distribution and engagement. Currently, direct imaging of neurodegenerative disease proteins in vivo is possible in humans only for amyloid and tau [123–125]. Most of the currently available amyloid PET ligands are limited in their utility because they bind only to insoluble fibrillar amyloid. Similarly, the development of tau tracers has challenged by the biochemical complexity and heterogeneity of tau deposits. Several promising tau tracers are now available for use in humans, but remain to be fully characterized with respect to their binding to specific isoforms and conformations of tau; in addition, all show significant off-target binding. The recent development of antibody-based PET ligands offers a potential solution to these issues. Such ligands have now been used to detect oligomeric Aβ in the mouse brain and this approach might also be used for α-synuclein and other neurodegenerative disease proteins for which no ligands are currently available [126].

A key issue that has arisen with respect to translatable biomarkers is lack of correlation between levels of disease proteins and functional outcomes in rodent models. For example, unlike humans, mouse AD models and some mouse HD models do not show neuronal cell death. However, even models that don’t show cell death do typically show neuronal dysfunction and synaptic loss. Imaging ligands that demonstrate synaptic loss are now being developed in humans, and could be reverse-translated for use in rodent studies [127]. Alternative translatable measures of neuronal function currently being explored include EEG [128] and functional imaging markers [129].

## Collaborating to accelerate therapeutic development

Until recently, drug discovery was the almost exclusive domain of biotechnology and pharmaceutical companies. Today, a new model for drug discovery has evolved, spurred in large part by initiatives led by patient advocacy groups, philanthropic organizations, the National Institutes of Health and other international funding agencies. This new model involves coordinated collaborations between academia, industry, private foundations, and government funding agencies, and incorporates patients and caregivers as key collaborators, knowledge resources, and decision-makers. Private foundations are increasingly taking on leadership roles that used to be handled primarily by government agencies, including the facilitation and scientific management of focused research initiatives, large-scale research consortia, and partnerships between academia, industry, and CROs.

Team approaches are particularly critical for the proteinopathies because of the heterogeneity and/or rarity of these conditions and the difficulty of recruiting sufficiently large patient cohorts for clinical trials for genetic disease. Patient advocacy groups and foundations are now playing critical roles in forcing some degree of standardization and scientific rigor, and providing critical natural history data for disease progression markers. Examples of such efforts include the Michael J. Fox Foundation Parkinson’s Progression Markers Initiative, the Target ALS drug discovery program, the Alzheimer’s disease Drug Discovery Foundation Access program, and the CHDI Foundation Preclinical Research program. Another key role for both governmental agencies and private foundations in supporting collaborations is through the development of public databases and “knowledge centers,” such as the National Alzheimer’s Coordinating Center and the Academic Drug Discovery Consortium. Recent national and international government initiatives supporting early drug discovery include the NIH Blueprint Neurotherapeutics Network and the European Innovative Medicines Initiative.

## Conclusion

There is active research ongoing to uncover the mechanisms by which disease-associated proteins misfold, aggregate, and cause cellular toxicity. Continued progress in our ability to interrogate amyloid-forming proteins and their interactions with other cellular proteins provide confidence that novel therapies will be identified for multiple disease states. Therapeutic options now being explored include targeting misfolded protein-chaperone interactions at various points in the proteostatic pathway, promoting protein clearance, and large-scale rebalancing of proteostatic network. However, the identification and in vivo validation of new therapeutic compounds is impeded by the shortage of known disease drivers and the lack of reliable biomarkers for monitoring therapeutic responses in relevant animal models. However, the increase in cooperative research and collaboration among the drug discovery community (pharmaceutical companies, foundations, academia, contract research organizations, clinicians, regulatory agencies, advocacy groups and patients) is a positive shift that can help accelerate the identification of novel therapeutic modalities.

## Abbreviations

AD: Alzheimer’s disease
ALS: Amyotrophic lateral sclerosis
Aβ: Beta amyloid
BACE: Beta amyloid cleaving enzyme
BBB: Blood brain barrier
CLIP: Chaperones linked to protein synthesis
CRO: Contract research organization
CSF: Cerebrospinal fluid
DNA: Deoxyribonucleic acid
GFP: Green fluorescent protein
HSP: Heat shock proteins
Htt: Ubiquitinated Huntingtin
IMS: Ion mobility spectrometry
IMS-MS: Ion mobility spectrometry-mass spectrometry
IPOD: Insoluble protein deposit
JUNQ: Juxtanuclear quality control
MS: Mass spectrometry
mTOR: Mammalian target of rapamycin
NIH: National Institutes of Health
NMR: Nuclear magnetic resonance
SDS-PAGE: Denaturing gel electrophoresis
TDP-43: TAR DNA binding protein-43
UPR: Unfolded protein response
UPS: Ubiquitin proteasome system
VCP: Valosin-containing protein VCP

## Footnotes

1 This review is based in part on an open scientific satellite symposium entitled “Protein Misfolding – Implications and Strategies” held in conjunction with the 45^th^ Annual Society for Neuroscience meeting in 2015, sponsored and organized by Charles River Laboratories. The meeting panelists included: Hyunsun Park (Health & Life Science Consulting), Judith Frydman (Stanford University), John Dunlop (Astra Zeneca), Marc Baumann (University of Helsinki), Alexander McCampbell (Biogen), Ron Kopito (Stanford University), Patrick Sweeney (Royal Veterinary College, University of London, Charles River Laboratories) and Robert Hodgson (Charles River Laboratories).
